# Resistance mechanism and proteins in *Aspergillus* species against antifungal agents

**DOI:** 10.1080/21501203.2019.1574927

**Published:** 2019-02-06

**Authors:** Sonia Kumari Shishodia, Shraddha Tiwari, Jata Shankar

**Affiliations:** Genomic Laboratory, Department of Biotechnology and Bioinformatics, Jaypee University of Information Technology, Solan, India

**Keywords:** Antifungal agents, proteomic, azole resistance, phytochemicals, Hsp70

## Abstract

**Aspergillus:**

species contain pathogenic and opportunistic fungal pathogens which have the potential to cause mycosis (invasive aspergillosis) in humans. The existing antifungal drugs have limitation largely due to the development of drug-resistant isolates. To gain insight into the mechanism of action and antifungal drug resistance in *Aspergillus* species including biofilm formation, we have reviewed protein data of *Aspergillus* species during interaction with antifungals drugs (polynes, azoles and echinocandin) and phytochemicals (artemisinin, coumarin and quercetin). Our analyses provided a list of *Aspergillus* proteins (72 proteins) that were abundant during interaction with different antifungal agents. On the other hand, there are 26 proteins, expression level of which is affected by more than two antifungal agents, suggesting the more general response to the stress induced by the antifungal agents. Our analysis showed enzymes from cell wall remodelling, oxidative stress response and energy metabolism are the responsible factors for providing resistance against antifungal drugs in *Aspergillus* species and could be explored further in clinical isolates. Also, these findings have clinical importance since the effect of drug targeting different proteins can be potentiated by combination therapy. We have also discussed the opportunities ahead to study the functional role of proteins from environmental and clinical isolates of *Aspergillus* during its interaction with the antifungal drugs.

**Abbreviations:**

IPA: invasive pulmonary aspergillosis; IA: invasive aspergillosis; AmB: Amphotericin B; CAS: Caspofungin; VRC: Voriconazole; ITC: Itraconazole; POS: Posaconazole; ART: Artemisinin; QRT: Quercetin; CMR: Coumarin; MIC: minimal inhibitory concentration

## Introduction

*Aspergilli* contain approximately 340 species that perform range of biological functions in the environment and about 40 of them are known to cause health problems (Samson et al. 2014; Thakur et al. 2015). *Aspergillus fumigatus, A. terreus, A. flavus, A. niger* and *A. oryzae* are among the most studied *Aspergilli* due to their medical, agricultural and industrial importance (Krijgsheld et al. 2013; Sugui et al. 2015). *Aspergillus oryzae* is widely used for traditional food fermentations in EastAsia, and *Aspergillus niger* is used to produce various enzymes (e.g. amylases and pectinases) and organic acids (Powell et al. 2013). Aspergillosis is a severe clinical problem caused by *Aspergillus* species, especially in immunocompromised patients, hence *Aspergilli* have emerged as important opportunistic fungal pathogens (Chowdhary et al. 2014). Also, a large number of patients have been diagnosed with chronic pulmonary aspergillosis worldwide following the treatment of pulmonary tuberculosis (Denning 2011). *Aspergillus* conidia are present in air, soil, food-products, indoor environment and plant debris. Conidia being smaller in size (2–5 µm) (Mousavi et al. 2016), are the main source for *Aspergilli* conidia distribution into the environment (Latgé 1999; Zmeili and Soubani 2007). Minuscule size of conidia enhances its existence in the air for a longer duration because of which it is inhaled by human beings and if these conidia are not cleared by phagocytic cells, may germinate into hyphae in respiratory mucosa (Zmeili and Soubani 2007). Conidia showed metabolically less active and possess prolonged viability in adverse conditions (Lamarre et al. 2008). *Aspergillus* causes invasive aspergillosis (IA) to extreme complications. Huge rise in drug-resistant isolates of *Aspergillus* species possess the additional threat to human beings (Hagiwara et al. 2016; Sanglard 2016). Currently, three class of antifungal drugs are commonly used for the treatment against *Aspergillus* mediated infections; polyenes, triazoles and echinocandin. These compounds target the cell wall either by disrupting ergosterol biosynthesis or β-1, 3-glucan or by targeting the ergosterol directly (Groll and Kolve 2004). We now have understood on how few of the existing antifungal drugs work. However, transcriptional factors and signalling cascades that are involved in providing antifungal drug resistance in *Aspergillus* isolates are partly known. Thus, we have reviewed proteins/enzymes that showed regulated expression during the interaction with antifungal agents in *Aspergillus* species.

## Present treatment strategies for IA

Treatment options have evolved which includes three major classes of antifungal viz. triazoles, echinocandin and polyenes. The triazoles include Itraconazole (ITC), VRC and POS. Isavuconazole is newly introduced azole against *Aspergillus* and approved against IA (Miceli and Kauffman 2015). Echinocandins include Caspofungin (CAS), Anidulafungin (AND) and micafungin (Supplementary file-1A). Treatment of IA using Amphotericin (AmB), possesses ergosterol binding tendency, forming fungicidal sterol sponge and disables membrane functions (Anderson et al. 2014). The major disadvantage of AmB is toxicity to humans. It has been observed that antifungal compounds showed adverse effects in children suffering from aspergillosis such as 3–5 mg/kg dosage of AmB per day for aspergillosis treatment causes infusion-related infection, hypokalemia and nephrotoxicity (Canadian Paediatric Society ID, UD ICA 2010). However, the liposomal AmB have shown the minimal toxic effect in IA patients and have prolonged persistence against azole-resistant *Aspergillus* species (Seyedmousavi et al. 2013). AmB is not recommended for aspergillosis caused by *A. terreus* and also AmB-resistant *Aspergillus* species, hence the combination therapy with a synergistic response is considered as alternative approach (Dannaoui et al. 2004; Elefanti et al. 2013). Antifungal combinational studies against *A. fumigatus* using *in vivo* or *in vitro*, and clinical methods have shown effective results (Ben-Ami et al. 2011; Stergiopoulou et al. 2011).

Triazoles are used as the more preferred choice of antifungals in clinical practices. Inhibition of ergosterol biosynthesis leads to the disruption of the structural unit of the cell membrane of fungi (Sanglard and Odds 2002). ITC, one of the triazoles, was the first drug introduced in azole class for aspergillosis patients in 1997 and other azoles evolved later. According to current status, azole resistance in *A. fumigatus* has emerged globally, thereby threatening the azole therapy against aspergillosis (Verweij et al. 2016). Van der Linden *et al*. have provided the mortality rate data in azole-resistant strains infected aspergillosis patients. Azoles resistant *Aspergillus* isolates were categorised on the basis of MIC values; ITC > 2 μg/ml, VRC > 2 μg/ml and posaconazole (> 0.5 μg/ml). These data were collected as per the guidelines mentioned in the CLSI reference method (van der Linden et al. 2011). Recent treatment strategies for azole-resistant as well as susceptible isolates of *A. fumigatus* are focused on the combinational drug therapy which includes azole (VRC) and echinocandin. Echinocandin inhibits cell wall biosynthesis by blocking the catalytic subunit of β-glucan synthase (Arendrup 2014).

According to a report by Ming Zhang 2014, *A. flavus* and *A. niger* mediated IPA was effectively treated by a combined effect of CAS and VRC. However, *A. fumigatus* mediated IPA treatment showed less efficacy in response to the combination of echinocandin and triazole (Krishnan-Natesan et al. 2012; Zhang et al. 2014). In a clinical report, 27.5% mortality rates were observed in monotherapy, whereas for combined (VRC and AND) therapy it was 19.3% (Marr et al. 2015). Denning, D.W. *et al*., reported that AmB- and azole-resistant *Aspergillus* isolates, a more frequently occurring clinical isolates, however, azoles are used as first-line therapy hence, these therapeutic options for IA need revision due to emergence of drug resistance *Aspergillus* isolates and intervention of novel therapeutic strategies to overcome this issue (Denning and Bowyer 2013). Isavuconazole is a recently approved drug (2015) for treatment of aspergillosis and mucormycosis, however as per the recent U.S. guidelines voriconazole has been recommended as a first-line therapy for aspergillosis (Misch and Safdar 2016).

The antifungal response is less effective when *A. fumigatus* forms a biofilm. Higher MIC of antifungal drug is required to destroy the biofilm structures, which has been one of the reason for drug resistance in *A. fumigatus* against the polyene, azole, and echinocandin (Mowat et al. 2008; Seidler et al. 2008). It has been hypothesised that extracellular matrix in biofilm confers drug resistance via absorbing antifungal molecules, thus disallowing their diffusion to the site of fungal cells. This has been supported by the formation of extracellular matrix that sequesters antifungal drugs and reduces drug susceptibility in *C. albicans* (Nett et al. 2010). Activation of multidrug resistance protein that pumps out antifungal drugs has been reported in biofilm structure. Thus, the role of efflux-pump in azole resistance has been observed (Rajendran et al. 2011) that possibly could be one of the reasons for treatment failure in aspergillosis cases.

Drug toxicity and pesticides exposure to humans are the major issues worldwide (Dorner 2004; Damalas and Eleftherohorinos 2011). Phytochemicals have acquired a lot of significance as a new candidate over the present drug discovery methods (Butler 2004) and are discussed in the later part of the review.

## Epidemiological pattern of drug resistance in *Aspergillus* species

Olga Rivero-Menendez recommended the need of antifungal susceptibility assay on the *Aspergillus* isolates derived from the clinical samples for the treatment of aspergillosis cases (Rivero-Menendez et al. 2016). In clinical practices, long-term use of azole drugs for aspergillosis is the major reason for the emergence of azole resistance (Hagiwara et al. 2016). Another route for the increase in azole resistance in *Aspergillus* is the extensive application of fungicides in the agriculture (Snelders et al. 2009; Chowdhary et al. 2013). Thus, investigations need to focus on to screen environmental samples that are resistant to azoles. Restricted use of azoles in agricultural, i.e. rotation of antifungal products, change in dosage and application periods could be the best possible strategy to reduce the burden of azole in the environment (Chowdhary et al. 2013; Berger et al. 2017). Additionally, some of the *Aspergillus* species has intrinsic resistant to certain antifungal and other species are susceptible to a certain class of drug but may become resistance due to the prolonged incomplete dosages of antifungal drugs. Recently, Rivero-Menendez *et al*. briefly showed the dominance and occurrence of azole drug resistance in *Aspergillus* species and reported the highest number of azole resistance isolates in European countries (Rivero-Menendez et al. 2016).

The occurrence of azoles resistant isolates of *A. fumigatus* varies from 6.6 to 28% worldwide. In the UK, it is 2.1–20%. In the Netherlands, Germany and France resistance level has up to 10–12% in clinical and environmental isolates. While in other continents (Asia, Africa, America and Australia) resistance level is also around 10% (Rivero-Menendez et al. 2016). Recently, 32.4% of *A. fumigatus* isolates in clinical samples were observed in India and out of the 1.75% were azole-resistant. Thus, lower occurrence of resistant isolates in India was observed in comparison to European countries that is probably the limited application of azole fungicides in Asia (Chowdhary et al. 2015; Directorate-General. ECHCP 2016).).

In general, resistant isolates are reported when MIC values are above the epidemiologic cut-off values based on these EUCAST defined breakpoints *Aspergillus* spp. (susceptible or resistant) for azoles (POS > 0.25 µg/ml, ISA > 1 µg/ml, ITC > 2 µg/ml and VRC > 2 µg/ml) (mEUCAST 2016). The Clinical and Laboratory Standards Institute has also defined MIC cut-off for various azoles viz. POS > 0.5 µg/ml, VRC > 1 µg/ml and ITC > 1 µg/ml (Espinel-Ingroff et al. 2010).

In addition, drug resistance in *Aspergillus* spp. is also mediated by the development of biofilms that provided temporary antifungal drug resistance and protects the pathogen in the hostile environment (Seidler et al. 2008; Villena et al. 2009; Bruns et al. 2010; Kaur and Singh 2014; Paul et al. 2017). In earlier studies, Mowat *et al*. showed the formation of biofilm structures in *A. fumigatus* cultures which are observed to be resistant to antifungal drugs (Mowat et al. 2007). Beauvais *et al*. also reported the extracellular matrix on the colony surface of *A. fumigatus* (Beauvais et al. 2007). Extracellular matrix helps hyphae to hold together to form biofilm structure, which permits reduced drug susceptibility. Also, major changes in metabolic activities have been observed during biofilm formation which might be associated with virulence (Muszkieta et al. 2013). Biofilm-associated infections have very high mortality rate and difficult to cure with existing drug therapies, thus further studies are needed to understand the role of biofilm in drug resistance.

*Aspergillus terreus* is another major cause of aspergillosis, reported in University and Hospital of Innsbruck in Austria and medical centres in Houston, Texas (Lass-Florl et al. 2007; Blum et al. 2008). As per the recent reports in India, 6.6% of *A. terreus* isolates were found among the aspergillosis cases in a referral Chest Hospital in Delhi (Chowdhary et al. 2015). In another study from India, it has been observed that only 8% of *A. terreus* isolates were susceptible to AmB with MICs (0.5–1 mg/L) and showed no particular genotypic pattern (Kathuria et al. 2015). Previously, it has been evident from previous reports that *A. terreus* isolates are naturally resistant to AmB (Steinbach et al. 2004a; Lass-Florl et al. 2007). Also, *A. terreus* has shown high *in vitro* and *in vivo* MICs for AmB which confirms the AmB resistance (Graybill et al. 2004; Steinbach et al. 2004b; Lass-Florl et al. 2005). Recently, failure of azole drug treatment against *A. terreus* has also been observed in Danish clinical samples by Arendrup *et al*. In their study (Arendrup et al. 2012), they have reported the development of ITC resistance in *A. terreus* which may be associated with M217I *Cyp51A* mutation.

*A. terreus* isolates (approximately 5%) showed resistance against posaconazole in *in-vitro* studies. High percentage (10%) posaconazole resistance *A. terreus s.s*. isolates were isolated from Austria, Germany and the UK (Zoran et al. 2018). Hence, lack of AmB response and azole (VRC) resistance has made *A. terreus* an infectious threat in immunocompromised patients (Pastor and Guarro 2014). As per another report from Alcazar-Fuoli, Laura *et al. A. niger* rarely showed varying MICs to ITC and the isolates which showed higher MIC for ITC also had higher MIC values to VRC and Isavuconazole compared with *A. fumigatus* MIC values (Alcazar-Fuoli et al. 2009). Higher MICs for other *Aspergillus* species such as *A. awamori* and *A. niger* have been observed in comparison to *A. tubingensis* (Szigeti et al. 2012). Additionally, biofilm formation has been reported in *A. niger* (Villena et al. 2009; Paul et al. 2017) probably accounting for high MIC against the drug.

*Aspergillus flavus* is mostly prevalent in arid climates and can tolerate extreme conditions and frequently occur in Africa, the Middle East and Southeast Asian countries (Krishnan et al. 2009). *A. flavus* is known to produce aflatoxins (potent carcinogen). This fungus contaminates various crops leads to economic losses in agriculture. Consumption of aﬂatoxin-contaminated foods or feeds causes severe illness in animal and humans such as aﬂatoxicosis, liver necrosis/liver cancer (Tiwari. 2018). Prevalence of *A. flavus* in India is about 45.4% and about 2.5% are resistant to VRC (Chowdhary et al. 2015; Sharma et al. 2018). In recent reports, some of the clinical isolates of *A. flavus* showed resistance to VRC and the high MICs linked to being T788G and Y319H alterations in the *cyp51C* gene (Liu et al. 2012; Paul and Rudramurthy 2015). Also, *Aspergillus alliaceus* (genomic similarity with *A. flavus*) showed high MICs value to AmB and echinocandins, which varies for different azoles (Balajee et al. 2007). From the above data, we could hypothesise that there is a large increase in the antifungal drug resistance from environmental and clinical *Aspergillus* isolates. Also, most of the antifungal agents are also used in crop protection and to preserve materials from fungal decay (van der Linden et al. 2015). Thus, it leads to the emergence of acquired resistance in this fungal species and there is a need for systematic surveillance programmes worldwide to reduce the use of antifungals in the environment.

## Proteomic approach to characterise the antifungal response in *Aspergilli*

Proteomic analysis has been applied to elucidate the resistant mechanism in resistant vs. susceptible strains and also in the identification of potential biomarkers (Vermeulen et al. 2018). Various research groups focused on the characterisation of *Aspergillus* spp. at development stages (Asif et al. 2006; Suh et al. 2012; Tiwari et al. 2016; Thakur and Shankar 2017). Comparative proteome analysis of resting conidia to mycelia provided biochemical and cellular pathway during the morphotypes of *Aspergilli* (Asif et al. 2006; Vodisch et al. 2009; Teutschbein et al. 2010; Tiwari et al. 2016; Thakur and Shankar 2017; Shankar et al. 2018). To date, proteomic-based analysis on how *Aspergilli* adapt to host condition has conceded information of *Aspergilli* infection mechanisms (Suh et al. 2012; Kubitschek-Barreira et al. 2013). To get a comprehensive picture of the response of drugs and phytochemical on *Aspergillus* spp., we have found limited reports in *Aspergilli* on proteome response under antifungal agents in case of drugs AmB, CAS and azoles (ITC and VRC) (Gautam et al. 2008, 2016; Cagas et al. 2011a; Amarsaikhan et al. 2017) and some of the phytochemicals like artemisinin (ART), coumarin-derivative (CMR) and quercetin (QRT) (Gautam et al. 2011; Singh et al. 2012; Tiwari and Shankar 2018b) which have been described. In the remaining part of the review, we discussed the major proteins and pathways involved during the exposure of drugs and phytochemicals.

### Proteome analysis in response to antifungal drugs

**Figure 1.**
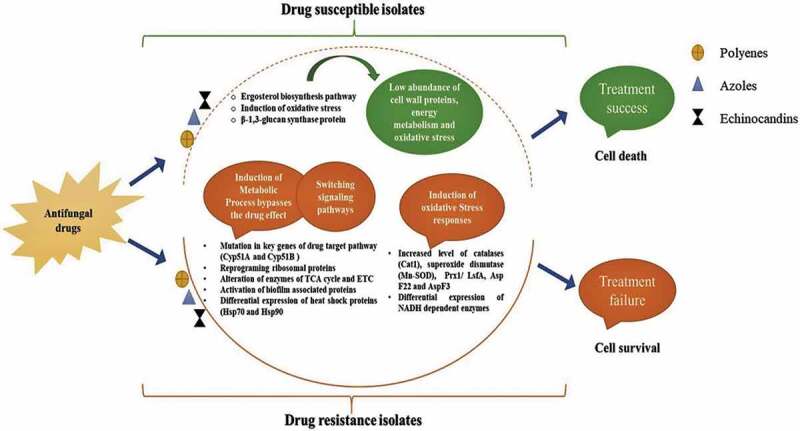
Probable determinants involved in the drug-resistance mechanism of *Aspergillus* species. Different colours of boxes represent antifungal drugs. In susceptible isolates, antifungal drugs targets cell wall, and generates oxidative stress. In resistance isolates, antifungal drugs showed the increased level of proteins from cell stress pathways and alternative metabolic pathways.

AmB, a class of polyene, acts via primarily binding to cell membrane ergosterol, thereby disrupting membrane function as well as ROS accumulation (Valiante et al. 2015). Gautam et al. (2008) studied the response of AmB in *A. fumigatus* and observed down-regulation of translation machinery and energy metabolism. Their study showed the abundance of 48 proteins using MALDI, 44 proteins were highly expressed and 4 of them showed less abundance. Additionally, ergosterol biosynthesis protein Erg13 (AFUA_3G10660) was up-regulated under AmB exposure (Gautam et al. 2008) Also, increased abundance of Hem13 (AFUA_1G07480), a heme biosynthetic protein on AmB exposure reflects the need of more heme-molecules; it acts as cofactor and prerequisite for the ergosterol biosynthesis. Most of the iron enzyme lost their activities on iron-deficiency (Shakoury-Elizeh et al. 2010). Induction of oxidative stress responses involves up-regulation of proteins such as catalase, manganese superoxide dismutase and Prx1/LsfA upon AmB treatment. Which further provide evidence that AmB damages cell due to oxidative stress (Gautam et al. 2008). ITC targets ergosterol leading to the accumulation of sterols that is toxic to the cells (Valiante et al. 2015). Gautam et al. (2016) also studied proteome of *A. fumigatus* under ITC stress that resulted in the differential abundance of 54 proteins. It has been observed that 12 proteins with the increased level of expression and 42 proteins with decrease in abundance (Gautam et al. 2016). The increased level of oxidative stress proteins like catalase, Cat1 were observed similar to AmB exposure, most abundant proteins in response to more than two antifungal agents are summarised in Table 1. Another study by Gautam et al. (2016) demonstrated the synergy of ITC with the ART, indicating a positive effect of this combination (Gautam et al. 2016). On the other hand, Cagas, Jain *et al*. studied the proteomic response of *A. fumigatus* against CAS using iTRAQ, at 24 and 48h and provided updated protein data set. Previously, they attempted to profile proteins at different morphotypes in *A. fumigatus* that study they provide differential protein expression patterns at various developmental stages in *A. fumigatus* (Cagas et al. 2011b) .Cagas *et al*., has observed 58 ribosomal proteins that are differentially expressed in their study, suggesting a shift in ribosomal programming in the cell. Also, in comparative study, ribosomal proteins at 24h post drug exposure of the susceptible strain was observed and only 4 out 19 proteins showed increased abundance in the resistant strain. Their results speculated a ribosomal reshuffling response to the CAS (Cagas et al. 2011a, 2011b). Gautam et al. (2008) observed that most of the ribosomal proteins in response to AmB were down-regulated in microarray data (Gautam et al. 2008). Interestingly, earlier reports suggested that mitochondrial hypoxia response domain protein (AFUA_1G12250) would be most promising biomarker which was down-regulated up to 16-fold at both 24 and 48h in susceptible strain and was relatively unaltered in the resistant strain under CAS but not observed during exposure of VRC and AmB to *A. fumigatus*, suggested as a biomarker specific to CAS (Gautam et al. 2008; Cagas et al. 2011a; Amarsaikhan et al. 2017) . From the above data, it is reflected that each drug molecule affects proteome of *Aspergilli* with a common target pathway. Some drugs may have different targets but broadly affects the common pathways which ultimately lead to cell cycle arrest. On the other hand, there is limited available protein data on biofilms in *Aspergillus* spp. We have observed that sets of protein are required for biofilm formation in *A. fumigatus*. Protein associated with the biofilms formation showed abundance in translational regulatory proteins (Muszkieta et al. 2013). Whereas, in the case of *A. niger* intracellular protein analysis of biofilm showed the 19% overexpressed and 32% differentially expressed protein spots when compared free-living submerged cultures using 2D-PAGE and MS-TOF analysis (Villena et al. 2009). Results suggested that proteins are involved in cell adhesion, which allow biofilm development and surface adhesion fermentation. Thus, from the discussed data available in Table 1 suggested that antifungal drugs mediated proteome response in *Aspergilli* majorly involves proteins that were found in managing the energy oxidative stress, alteration of cell wall biosynthesis and ribosomal reprogramming. Also, induction of bypass energy metabolism pathways is evident upon exposure to all the antifungal drugs Figure 1. These pathways and proteins might be involved in a resistant mechanism and may also be explored as new drug targets in drug resistance *Aspergilli*.

**Table 1.** Most abundant proteins in-response to more than two antifungal agents in *Aspergillus* species through proteomic studies.

			Antifungal drugs				Phytochemicals		
S. no.	Name of the protein	Name of organisms	AmB (24h) (Gautam et al. 2008)	CAS (24h & 8h)(Cagas et al. 2011a)	VRC (4h) (Amarsaikhan et al. 2017)	ITC (24) (Gautam et al. 2016)	ART (3h) (Gautam et al. 2011)	QRT (7h) (Tiwari and Shankar 2018b)	CMR (16h) (Singh et al. 2012)
1	Mitochondrial Hsp70 chaperone (Ssc70), putative	*Aspergillus fumigatus, Aspergillus flavus*	+	−	+	+	−	+	+
2	Enolase	*Aspergillus fumigatus*	+	−	+	+	−	−	+
3	Ubiquinol-cytochrome C reductase complex core protein 2)	*Aspergillus fumigatus*	−	_+_	+	−	_+_	−	+
4	Glutamate/Leucine/Phenylalanine/Valine dehydrogenase, putative	*Aspergillus fumigatus*	+	−	+	+	−	−	+
5	Glutamate carboxypeptidase, putative	*Aspergillus fumigatus, Aspergillus flavus*	−	−	+	+	−	+	+
6	Allergen Asp F3	*Aspergillus fumigatus*	−	+	+	+	−	−	+
7	Thioredoxin (Thioredoxin TrxA	*Aspergillus fumigatus*	+	−	+	+	−	−	+
8	Phosphoglycerate kinase	*Aspergillus fumigatus*	+	+	−	+	−	−	+
9	NAD-dependent formate dehydrogenase	*Aspergillus fumigatus*	+	+	−	+	−	−	+
10	Mycelial catalase Cat1	*Aspergillus fumigatus, Aspergillus flavus*	+	+	−	+		+	−
11	Fumarate hydratase	*Aspergillus fumigatus*	+	−	+	+	−	−	−
12	Cobalamin-independent methionine synthase MetH	*Aspergillus fumigatus*	−	+	+	−	−	−	+
13	NADH-ubiquinone oxidoreductase 213 kDa subunit	*Aspergillus fumigatus*	−	−	+	−	+	−	+
14	Translation elongation factor EF-2 subunit, putative	*Aspergillus fumigatus*	+	−	+	−	−	−	+
15	Antigenic mitochondrial protein HSP60, putative	*Aspergillus fumigatus*	−	+	+	−	−	−	+
16	Aminopeptidase P, putative	*Aspergillus fumigatus, Aspergillus flavus*	−	−	+	−	−	+	+
17	Autophagic serine protease Alp2	*Aspergillus fumigatus*	−	−	+	+	−	−	_+_
18	Proteasome regulatory particle subunit (RpnL), putative	*Aspergillus fumigatus*	+	−	+	−	−	−	+
19	Conidial hydrophobin RodB	*Aspergillus fumigatus*	+	−	+	−	+	−	−
20	1,3-beta-Glucanosyltransferase Bgt1	*Aspergillus fumigatus, Aspergillus flavus*	−	−	+	−	+	+	
21	Cobalamin-independent methionine synthase MetH/D	*Aspergillus fumigatus*	−	+	+	−	−	−	+
22	Integral membrane protein	*Aspergillus fumigatus, Aspergillus flavus*	+	+	−	−	−	+	−
23	Antioxidant protein LsfA	*Aspergillus fumigatus*	+	+	−	−	−	−	+
24	Malate dehydrogenase, NAD-dependent	*Aspergillus fumigatus*	+	−	−	+	−	−	+
25	ATP synthase proteolipid P2, putative	*Aspergillus fumigatus*	+	+	−	−	+	−	−
26	Aldehyde reductase	*Aspergillus fumigatus*	+	−	−	+	−	−	+

Time-points for drug treatment has been mentioned against the antifungal agents, “(+) represents the presence of the protein”, “(−) represents the absence of the protein”

### Proteome analysis in response to phytochemicals

Natural plant products with antifungal properties may offer a safe and effective alternative treatment strategy against aspergillosis. As of now the antimicrobial activity of these compounds, even studied comprehensively, still, none of them is available for practice at the clinical level. Screening of phytochemicals such as ART and CMR as anti-*Aspergillus* agents have been carried out (Gautam et al. 2011; Singh et al. 2012). Recently, we explored the anti-*Aspergillus* property of QRT along with other phytochemicals, QRT showed the strongest activity with MIC_50_ at 36 µg/ml in *A. parasiticus* and 113 µg/ml in toxigenic *A. flavus* (Tiwari et al. 2017). In addition, proteome response under QRT exposure showed the abundance of proteins of oxidative stress, cell wall proteins and membrane transport activity (influx and efflux proteins). Also under QRT stress switching of the signalling from MAPK to cAMP/PKA in *A. flavus* has been observed (Tiwari and Shankar 2018b). The similar kind of trend in signalling pathway was observed in *Saccharomyces cerevisiae* and *Candida albicans*, a remarkable decrease in PKC signalling was noted. In case of CAS drug treatment which involves drug resistance through calcineurin function, the activation of downstream MAPK pathway was also noted (LaFayette et al. 2010). In addition, HPLC analysis of *A. flavus* conidia grown for 48 h time point under influence of QRT showed a significant decrease in production of AFB1 (up to 51%) (Tiwari and Shankar 2018b). Gautam et al. (2008) investigated the exposure of ART to *A. fumigatus* using MALDI-ToF/ToF that leads to the differential abundance of 85 proteins; 29-increased and 56-decreased. Decreased expression of proteins included conidial hydrophobin (RodB), thaumatin domain protein (PhiA), galactomannan protein indicating remodelling of the cell wall, similar to AmB exposure (Gautam et al. 2008). Also, down-regulation of two mitochondrial genes, NADH dehydrogenase and NADH-ubiquinone oxidoreductase in microarray data and absence in protein dataset under ART exposure, reflects ART targets NADH dehydrogenase of *A. fumigatus* (Gautam et al. 2011). It was supported by the earlier study that deletion of NADH gene in yeast confers more resistance to ART, whereas overexpression of the NADH gene leads to more susceptibility in the yeast (Li et al. 2005). However, most of the other genes related to oxidative phosphorylation pathway showed up-regulation under ART exposure. As, it has been reported by Gautam et al. (2011) that ART may disrupt the membrane potential. Up-regulated transcripts may allow to re-established membrane potential by over-expressing these genes belonging to this pathway (Gautam et al. 2011). Importantly, the oxidative phosphorylation pathway was not affected significantly in *C. albicans, S. cerevisiae* and *A. fumigatus* (Zhang et al. 2002; Agarwal et al. 2003; Liu et al. 2005; Da Silva Ferreira et al. 2006; Yu et al. 2007; Gautam et al. 2008) upon standard antifungal drug treatment.

Subsequently, Singh *et al*., provided the proteome profile in *A. fumigatus* under exposure to synthetic coumarin-derivatives (SCD-1) and demonstrated the antifungal mechanism. Previously, Singh *et al*. showed SCD-1 a potent inhibitor of pathogenic *Aspergilli* at MIC_90_ of 15.62 μg/ml. In their study, differential abundance of 143 proteins was observed; 96-up and 30-down. Also, 4 proteins in control alone and 13 proteins in SCD-1 treated sample were reported. Proteins, involved in riboflavin biosynthesis fall under the category of decreased abundance, have been suggested as a novel target of SCD-1 (Singh et al. 2012). The proteomic analysis of antifungal treated *A. fumigatus* and *A. flavus* indicated that the different targets with similar or different altered pathways. Whereas there is no such proteomic data on *A. terreus* and *A. niger* in response to antifungal drugs. Researcher attempted to profile proteomic data on various morphotypes and at various stress conditions in *A. terreus* and *A. niger* (Sorensen et al. 2009; Lu et al. 2010; Thakur and Shankar 2017) and recently it has been reviewed (Shankar et al. 2018) on underlying mechanism to exit conidial dormancy in *Aspergillus* species.

### Most abundant proteins under the exposure of both antifungal drug and phytochemicals

Proteins involved in cell stress viz. Aspf3 and enolase (glycolytic enzyme) which were frequently seen under the exposure of all drugs (AmB, VRC, CAS, ITC) and CMR (Gautam et al. 2008, 2016; Singh et al. 2012; Amarsaikhan et al. 2017). iTRAQ analysis was performed to check the effect of CAS on the expression level of Aspf3 at 48 h time point in *A. fumigatus* susceptible strain showed an increase of 3.5-fold and the resistant strain showed a decrease of 1.5-fold (Cagas et al. 2011a). Aspf3 encodes, a thioredoxin peroxidase, and an increase in the expression has been reported in response to hydrogen peroxide oxidative stress (Lessing et al. 2007; Cagas et al. 2011a) which clearly indicate that CAS mediated oxidative injury can easily cope up in resistant strains. Differential expression of a variety of antioxidant enzymes and enzymes of carbohydrate metabolism indicate the sensitivity of these metabolic pathways to antifungals. In case of oxidative stress mycelial catalase (Cat1) and aldehyde reductase was reported which induces oxidative stress, revealed that drugs mediate damage of fungal cell membrane resulting in oxidative stress conditions via ROS activation. Whereas, antifungal agents induce the proteins of regulation of ROS homeostasis significantly (Cowen and Lindquist 2005; Blum et al. 2013; Jukic et al. 2017) validate the existing reports on the role of oxidative stress response in drug resistance studies.

Enolase (Aspf 22) has been reported an allergen which stimulates a strong IFNγ immune response in humans and its homolog in *C. albicans* showed partial protection as a vaccine candidate (Denikus et al. 2005; Chaudhary et al. 2010). Presence of enolase (involved in energy metabolism) in hyphae of *Aspergilli* suggests that this enzyme may facilitate tissue invasion in the host (Denikus et al. 2005; Moloney et al. 2016; Shankar et al. 2018). In *A. fumigatus*, enolase was abundantly expressed under the influence of antifungal drug suggesting energy metabolism is vital to overcoming the drug stress. We also observed heat shock protein Hsp70 was overexpressed during antifungal drug treatment. The role of other heat shock proteins in response to an antifungal drug has been recently discussed (Cowen and Lindquist 2005; Blatzer et al. 2015; Tiwari et al. 2015; Tiwari and Shankar 2018a). Various enzymes of the glycolytic pathway, TCA cycle and electron transport chain were abundant under antifungal agent exposure (Table 1). Similarly, the abundance of TCA cycle proteins has also been observed in the proteome of biofilm of *A. fumigatus* that may contribute towards the persistence of the organism inside the host (Muszkieta et al. 2013). These results reflect a shift in energy metabolism to glyoxylate cycle under antifungal and biofilm conditions to combat the shortage of energy. Thus, up-regulation of TCA cycle proteins under antifungal stress may suggest their role in resistance mechanism in *Aspergilli*. Under the exposure of CAS and AmB, ribosomal proteins were found to be highly expressed, which indicates drug-mediated ribosomal reshuffling (Gautam et al. 2008; Cagas et al. 2011a). It reflects the requirement of more protein synthesis under antifungal stress to overcome the inhibitory effects of antifungal agents. Proteasome regulatory protein, RpnL (Gautam et al. 2008; Singh et al. 2012; Amarsaikhan et al. 2017) was also observed under the influence of antifungal agents (AmB, VRC and CMR).

The proteins/enzymes which are commonly targeted by antifungals are of great interest to increase the efficacy of treatment by using it in combination therapy. After reviewing the existing literature, we have compiled a dataset with similar proteins under the antifungal stress in different *Aspergillus* species (Supplementary file-1B). Out of 72 most abundant proteins, 26 proteins belonging to different metabolic pathways showed higher abundance underexposure of more than two antifungal agents (Table 1) whereas nine proteins are specific to one antifungal agent and remaining showed differential presence in antifungal agents. Most of these antifungal agents targeted the metabolism of the cell wall, ergosterol and oxidative stress proteins.

## Insight into the drug-resistance mechanism in *Aspergillus* species

Antifungal drugs used for the treatment of various forms of aspergillosis face challenges due to the development of resistance in *Aspergillus*. Also, the prolonged use of the antifungal drug is one of the major causes of acquired drug resistance. It also depends on the type of *Aspergillus* species, antifungal drugs as well as on the geographical location. Though, the data on drug-resistance genes and mutations in the genome are available, but therapeutic choices are limited that make it difficult to control the invasive secondary fungal infections.

The major categories of mechanism of drug resistance include (1) *Alterations in drug targets*, due to mutations in target which reduces binding of drug to the target, (2) *loss in drug efficacy*, due to increase in drug efflux, overexpression of drug targets and sequester of antifungal agents and (3) *Metabolic bypass*, involves the activation of compensatory mechanisms which nullifies the toxic effects exerted by antifungals (Sanglard 2016). More recently, different resistance patterns with new mechanisms were observed, including intrinsic resistance in *Aspergillus* spp. and the emergence of simultaneous resistance to more than one class of drugs. Further, to increase the efficacy of existing drugs (combination of drugs) and through targeted drug therapy could be the future. The occurrence of drug resistance in *Aspergilli* and known mechanism of resistance of standard antifungal drugs are summarised in (Supplementary file-1A).

*Aspergillus fumigatus* and *A. terreus* are most extensively studied in the clinical spectrum due to the presence of high level of resistant isolates in comparison to other *Aspergillus* species. The major targets of the azoles reported by Mellado, E *et al*., are *cyp51A* and *cyp51B* (Cyp51 proteins (Mellado et al. 2001). These two encoded by different genes sharing 63% of sequence identity. Azole-resistant strains of *A. fumigatus* contain point mutations or overexpress *cyp51A* to provide resistance. The *cyp51A* encodes 14-sterol-demethylase, ergosterol is one of the major constituents of fungal cell structure (Snelders et al. 2010). Triazoles interact with the active site of Cyp51A, thus, hinder the ergosterol biosynthesis. Fungal cell death occurs due to the alteration in membrane fluidity (Snelders et al. 2009; Chowdhary et al. 2014). The wide use of other azole fungicides in agricultures exhibited a similar molecular structure to medical triazoles causing the evolution of cross-resistance in clinical practice (Snelders et al. 2012). Hagiwara et al. recently reviewed mutation at different sites in the cyp51 genes or tandem repeats in promoter region (TR34/L98H and TR46/Y121F/T289A) in *Aspergilli* contributing to resistance against azoles (Hagiwara et al. 2016). The whole genome sequencing of azole-resistant and susceptible (clinical and environmental) strains of *A. fumigatus* (from India, Netherlands and UK) have revealed that the environmental route is dominating with mutations in the cyp51A gene (TR34/L98H) in providing azole resistance (Abdolrasouli et al. 2015). On the other side, mutant study on biofilms of *A. fumigatus*, it has been observed that glycophosphatidylinositol-anchored cell wall protein (*cspA*) plays role in biofilm development, cell wall integrity and affects the drug-response (Fan et al. 2015). In addition, the substitution of S678P in Fks1p, the major subunit of glucan synthase, imparts resistance against echinocandin in *Aspergillus fumigatus* (Rocha et al. 2007). Thus, the data suggested that *Alterations in drug targets* is the most common strategy for resistance against antifungal drugs in *Aspergilli*.

*A. terreus* has been observed to be an intrinsic resistant to AmB in comparison to other *Aspergilli*, however, underlying molecular machinery is less unclear. The up-regulation of ergosterol biosynthesis genes (ERG5, ERG6 and ERG25) has been suggested to provide resistance against AmB (Walsh et al. 2003; Barker et al. 2004; Deak et al. 2009). Whereas, another study suggested that ergosterol content in *A. terreus* may have a little role in providing resistance against AmB. They further added AmB resistance *Aspergillus* strains absorb less amount of AmB drug, and also showed better protection management against oxidative damage due to the drug in *A. terreus* resistance isolates (Blum et al. 2013). According to a recent report in AmB resistance strains, SOD activity was more in comparison to susceptible isolates in *Aspergilli* (Jukic et al. 2017). Superoxide dismutase detoxifies superoxide anions, which are the precursor of ROS. Also, high production of gliotoxin (redox-active-metabolite) in biofilms of *A. fumigatus* was observed which confers resistance by enabling its growth and persistence in the host tissue (Bruns et al. 2010).

Another important key protein Hsp70 could be the regulator of AmB resistance, Blatzer *et al*. Use of inhibitors of Hsp70 and Hsp90, significantly increase the efficacy of AmB and azole drugs in resistant *A. terreus* isolates (Cowen and Lindquist 2005; Blatzer et al. 2015; Tiwari et al. 2015). However, the mechanisms by which heat shock proteins involved in drug resistance needs to be fully investigated. It primarily suggested that *Metabolic bypass* is the common strategy for the AmB resistance in *A. terreus*. Protein level analysis of host and pathogen under antifungal exposure may reveal the role of enzyme and protein which will be further investigated for their role in signalling and stress pathways. Thus, the advancement of existing technologies to fill the research gap is the challenges ahead.

## Future aspects and conclusion

The mechanisms contributing to drugs-resistance include reducing drug-target interactions by increasing/decreasing expression of proteins involved in cell wall modulation, oxidative stress, heat shock proteins and energy metabolism. Also, other modes such as redox imbalance, ROS homeostasis and alteration in membrane fluidity contribute to the drug resistance. Another strategy of antifungal drug resistance includes biofilm formation, which allows adhesion of fungal cells on host surface. Similarly, efforts have been made to identify the specific protein in response to antifungal drugs that include overexpression of Cat1, Prx1/LsfA, enolase, thioredoxin peroxide (Aspf 3), Sod2. Fewer proteins enlisted are less abundant belonged to cytochrome C, RodA, PhiA. The major targets of the azoles are Cyp51A and Cyp51B. Hsp70 and Hsp90 contribute to protect cells during stress conditions. Proteins such as Sod2, Cat1, thioredoxin peroxide, Hsp70 and Hsp90 could be explored further as targets in resistant *Aspergillus* isolates. Following the facts that the level of certain protein was changed under drug exposure does not really mean its involvement in the drug-resistant mechanism, but provide opportunities that it can be tested by comparing proteome from drug-susceptible and resistant *Aspergillus. Alterations in drug targets* and *metabolic bypass* are common strategy for resistance against antifungal drugs in *Aspergilli*. Further, expression and mutation studies are required to understand the exact resistance pattern of these probable resistance determinants in *Aspergilli*. Synergistic drug combinations affecting different targets could provide an effective and alternate treatment strategy against drug-resistance fungal pathogens in immunocompromised patients. Furthermore, advances in the proteomic analysis of clinical vs. environmental isolates of *Aspergillus* may add detail insight into the drug-resistance mechanism.

## Supplementary Material

Supplemental Material
