# Supplementary material for: Resistance mechanism and proteins in Aspergillus species against antifungal agents
Source: Mycology. 2019 Feb 6;10(3):151–65. doi: 10.1080/21501203.2019.1574927 (PMC6691784; doi:10.1080/21501203.2019.1574927)
Supplement: Supplemental Material [file TMYC_A_1574927_SM2226.docx]

**Supplementary file-1A**

**Mechanism of resistance in *Aspergilli* against standard antifungal drugs**

| **S.no.** | **Class of drugs** | **Resistant Aspergillus species** | **Targets of drug** | **Mechanism of resistance** | **References** |
| --- | --- | --- | --- | --- | --- |
| 1. | Polyenes  AmB | *Aspergillus terreus,*  *Aspergillus fumigatus, Aspergillus flavus* | Ergosterol and oxidative stress | Less or no membrane ergosterol content due to the mutation in *ERG3*or *ERG6*  Increased activity of catalase and heat shock proteins | ([Sanglard D. and Odds 2002](#_ENREF_83); [Anderson et al. 2014](#_ENREF_4); [Cuenca-Estrella 2014](#_ENREF_27); [Srinivasan et al. 2014](#_ENREF_94)) |
| 2. | Azoles  Fluconazole Itraconazole Voriconazole Posaconazole ) | *Aspergillus fumigatus, Aspergillus flavus* | Cyp51 proteins, block demethylation of C-14 of lanosterol | Mutation or overexpression of Cyp51gene | ([Sanglard D. and Odds 2002](#_ENREF_83); [Cuenca-Estrella 2014](#_ENREF_27); [Srinivasan et al. 2014](#_ENREF_94); [Rivero-Menendez et al. 2016](#_ENREF_79)) |
| 3. | Echinocandin  (Caspofungin Micafungin Anidulafungin) | *C. glabrata, A. fumigatus* | β‑1,3‑glucan synthase protein | Mutations in fks1 and raised chitin levels | ([Perlin 2007](#_ENREF_77); [Rocha et al. 2007](#_ENREF_80); [Nussbaum et al. 2010](#_ENREF_73); [Arendrup 2014](#_ENREF_5); [Cuenca-Estrella 2014](#_ENREF_27); [Srinivasan et al. 2014](#_ENREF_94)) |

**Supplementary file 1B: Protein/enzymes expressed in *Aspergillus* spp. in response to antifungal (drugs/phytochemicals) stress observed inone ormore than one case.**

| **SNo.** | **Protein** | **Source** | **Drugs** | | | | **Phytochemicals** | | |
| --- | --- | --- | --- | --- | --- | --- | --- | --- | --- |
|  |  |  | **Polyene** | **Echinocandin** | **Azole** | | **Artemisinin**  ([Gautam, Upadhyay et al. 2011](#_ENREF_5)) | **Quercetin**  (Shraddha et.al 2018) | **Coumarin**  SCD-1  ([Singh, Gupta et al. 2012](#_ENREF_6)) |
|  |  |  | **AmphotericinB**  ([Gautam, Shankar et al. 2008](#_ENREF_4)) | **Caspofungin**  ([Cagas, Jain et al. 2011](#_ENREF_2)) | **Voriconazole**  ([Amarsaikhan, Albrecht-Eckardt et al. 2017](#_ENREF_1)) | **Itraconazole**  ([Gautam, Mushahary et al. 2016](#_ENREF_3)) |  |  |  |
| 1 | Phosphoglycerate mutase, 2,3-bisphosphoglycerate-independent | *Aspergillus fumigatus* | - | - | + | + | - | - | - |
| 2 | Enolase | *Aspergillus fumigatus* | + | - | + | + | - | - | + |
| 3 | *Succinyl-CoA synthetase beta subunit, putative | *Aspergillus fumigatus* | - | - | + | - | - | - | - |
| 4 | Fumarate hydratase | *Aspergillus fumigatus* | + | - | + | + | - | - | - |
| 5 | Transaldolase | *Aspergillus fumigatus* | - | - | + | - | - | - | - |
| 6 | Cobalamin-independent methionine synthase MetH | *Aspergillus fumigatus* | - | + | + | - | - | - | + |
| 7 | Choline oxidase (CodA), putative | *Aspergillus fumigatus* | - | + | + | - | - | - | - |
| 8 | Ubiquinol-cytochrome C reductase complex core protein 2) | *Aspergillus fumigatus* | - | _+_ | + | - | _+_ | - | + |
| 9 | NADH-ubiquinone oxidoreductase 213 kDa subunit | *Aspergillus fumigatus* | - | - | + | - | + | - | + |
| 10 | Outer mitochondrial membrane protein porin | *Aspergillus fumigatus* | - | + | + | - | - | - | - |
| 11 | *Translation elongation factor eEF-3, putative | *Aspergillus fumigatus* | - | - | + | - | - | - | - |
| 12 | Translation elongation factor EF-2 subunit, putative | *Aspergillus fumigatus* | + | - | + | - | - | - | + |
| 13 | *G-protein complex beta subunit CpcB | *Aspergillus fumigatus* | - | - | + | - | - | - | - |
| 14 | Assimilatory sulfite reductase | *Aspergillus fumigatus* | - | - | + | - | - | - | _+_ |
| 15 | Glutamate/Leucine/Phenylalanine/Valine dehydrogenase, putative | *Aspergillus fumigatus* | + | - | + | + | - | - | + |
| 16 | Uridylate kinase Ura6 | *Aspergillus fumigatus* | - | - | + | - | - | + | - |
| 17 | RNP domain protein | *Aspergillus fumigatus* | + | - | + | - | - | - | - |
| 18 | Mitochondrial Hsp70 chaperone (Ssc70), putative | *Aspergillus fumigatus,* | + | - | + | + | - | + | + |
| 19 | Antigenic mitochondrial protein HSP60, putative | *Aspergillus fumigatus* | - | + | + | - | - | - | + |
| 20 | Aminopeptidase P, putative | *Aspergillus fumigatus, Aspergillus flavus* | - | - | + | - | - | + | + |
| 21 | Glutamate carboxypeptidase, putative | *Aspergillus fumigatus, Aspergillus flavus* | - | - | + | + | - | + | + |
| 22 | Carboxypeptidase 3 (Carboxypeptidase CpyA/Prc1, putative | *Aspergillus fumigatus* | - | - | + | - | - | - | + |
| 23 | Autophagic serine protease Alp2 | *Aspergillus fumigatus* | - | - | + | + | - | - | + |
| 24 | *Ubiquitin C-terminal hydrolase L3 | *Aspergillus fumigatus* | - | - | + | - | - | - | - |
| 25 | Proteasome regulatory particle subunit (RpnL), putative | *Aspergillus fumigatus* | + | - | + | - | - | - | + |
| 26 | Conidial hydrophobinRodB | *Aspergillus fumigatus* | + | - | + | - | + | - | - |
| 27 | *Thiamine biosynthesis protein (Nmt1), putative | *Aspergillus fumigatus* | - | - | + | - | - | - | - |
| 28 | *Pyridoxine biosynthesis protein | *Aspergillus fumigatus* | - | - | + | - | - | - | - |
| 29 | 1,3-beta-Glucanosyltransferase Bgt1 | *Aspergillus fumigatus, Aspergillus flavus* | - | - | + | - | + | + | - |
| 30 | *Mitochondrial peroxiredoxin Prx1, putative | *Aspergillus fumigatus* | - | - | + | - | - | - | - |
| 31 | Allergen Asp F3 | *Aspergillus fumigatus* | - | + | + | + | - | - | + |
| 32 | *Allergen, putative | *Aspergillus fumigatus* | - | - | + | - | - | - | - |
| 33 | L-PSP endoribonuclease family protein (Hmf1), putative | *Aspergillus fumigatus* | - | + | + | - | - | - | - |
| 34 | Thioredoxin (Thioredoxin TrxA | *Aspergillus fumigatus* | + | - | + | + | - | - | + |
| 35 | HAD superfamily hydrolase, putative | *Aspergillus fumigatus* | - | - | + | - | - | - | + |
| 36 | ThiJ/PfpI family protein) | *Aspergillus fumigatus* | - | + | + | - | - | - | - |
| 37 | *Dienelactone hydrolase family protein | *Aspergillus fumigatus* | - | - | + | - | - | - | - |
| 38 | Cell wall glucanase Crf1 | *Aspergillus fumigatus* | - | + | - | - | + | - | - |
| 39. | Phosphoglycerate kinase | *Aspergillus fumigatus* | + | + | - | + | - | - | + |
| 40 | Cell wall protein PhiA | *Aspergillus fumigatus* | - | + | - | - | _+_ | - | - |
| 41 | Cobalamin-independent methionine synthase MetH/D | *Aspergillus fumigatus* | - | + | + | - | - | - | + |
| 42 | Ubiquinol-cytochrome C reductase complex core protein 2, putative | *Aspergillus fumigatus* | - | + | + | - | - | - | + |
| 43 | 14-3-3 family protein | *Aspergillus fumigatus* | - | + | - | - | - | - | + |
| 44 | Nucleoside diphosphate kinase | *Aspergillus fumigatus* | + | + | - | - | - | - | - |
| 45 | Conserved hypothetical protein | *Aspergillus fumigatus* | - | + | - | + | - | - | + |
| 46 | Sur7 protein | *Aspergillus fumigatus* |  | + | - | - | + | - | - |
| 47 | Siderochrome-iron transporter, putative | *Aspergillus fumigatus* | + | + | - | - | - | - | - |
| 48 | Integral membrane protein | *Aspergillus fumigatus, Aspergillus flavus* | + | + | - | - | - | + | - |
| 49 | Antioxidant protein LsfA | *Aspergillus fumigatus* | + | + | - | - | - | - | + |
| 50 | GPI anchored protein, putative | *Aspergillus fumigatus* | - | + | - | - | + | - | - |
| 51 | Extracellular thaumatin | *Aspergillus fumigatus* | - | + | - | - | + | - | - |
| 52 | Fructose-bisphosphate aldolase, class II | *Aspergillus fumigatus* | - | + | - | - | - | - | + |
| 53 | Cell wall glucanase, putative | *Aspergillus fumigatus* | - | + | - | - | + | - | - |
| 54 | NAD-dependent formate dehydrogenase | *Aspergillus fumigatus* | + | + | - | + | - | - | + |
| 55 | Mn Superoxide dismutase | *Aspergillus fumigatus* | + | + | - | - | - | - | - |
| 56 | Mycelial catalase Cat1 | *Aspergillus fumigatus, Aspergillus flavus* | + | + | - | + | - | + | - |
| 57 | Coproporphyrinogen III oxidase | *Aspergillus fumigatus* | + | - | - | - | + | - | - |
| 58 | Malate dehydrogenase, NAD-dependent | *Aspergillus fumigatus* | + | - | - | + | - | - | + |
| 59 | ATP synthase proteolipid P2, putative | *Aspergillus fumigatus* | + | + | - | - | + | - | - |
| 60 | Ornithine aminotransferase | *Aspergillus fumigatus* | + | - | - | + | - | - | - |
| 61 | Aldehyde reductase | *Aspergillus fumigatus* | + | - | - | + | - | - | + |
| 62 | Glycerol dehydrogenase (GldB), putativ | *Aspergillus fumigatus* | + | - | - | + | - | - | + |
| 63. | Methyltransferase SirN-like | *Aspergillus fumigatus* | + | - | - | + | - | - | - |
| 64. | Sterol 24-C-methyltransferase (ERG 6) | *Aspergillus fumigatus* | + | - | - | - | + | - | - |
| 65 | Fasciclin domain family | *Aspergillus fumigatus* | + | - | - | - | + | - | - |
| 66 | Thioredoxin reductase glit | *Aspergillus fumigatus* | - | - | - | + | - | - | + |
| 67 | Lysophospholipase Plb3 | *Aspergillus fumigatus* | - | - | - | + | + | - | - |
| 68 | Glycerol dehydrogenase Gcy1 | *Aspergillus fumigatus* | - | - | - | + | - | - | + |
| 69 | NADH-cytochrome b5 reductase | *Aspergillus fumigatus* | - | - | - | - | + | - | + |
| 70 | Glycosyl transferase | *Aspergillus fumigatus, Aspergillus flavus* | - | - | - | - | + | + | - |
| 71 | Fatty acid synthase | *Aspergillus fumigatus, Aspergillus flavus* | - | - | - | - | + | + | - |
| 72 | C2H2 transcription | *Aspergillus fumigatus,*  *Aspergillus flavus* | - | + | - | - | - | + | - |

* Proteins those are specific to antifungal response, ‘(+) represents the presence of the protein’, ‘(-) represents the absence of the protein’
